# Interviewer versus self-administered health-related quality of life questionnaires - Does it matter?

**DOI:** 10.1186/1477-7525-9-30

**Published:** 2011-05-10

**Authors:** Milo A Puhan, Alka Ahuja, Mark L Van Natta, Lori E Ackatz, Curtis Meinert

**Affiliations:** 1 Department of Epidemiology, Johns Hopkins Bloomberg School of Public Health, Baltimore, MD, USA; 2 Department of Ophthalmology, Northwestern Medical Faculty Foundation, Chicago, IL, USA

**Keywords:** AIDS, quality of life, questionnaire, administration

## Abstract

**Background:**

Patient-reported outcomes are measured in many epidemiologic studies using self- or interviewer-administered questionnaires. While in some studies differences between these administration formats were observed, other studies did not show statistically significant differences important to patients. Since the evidence about the effect of administration format is inconsistent and mainly available from cross-sectional studies our aim was to assess the effects of different administration formats on repeated measurements of patient-reported outcomes in participants with AIDS enrolled in the Longitudinal Study of Ocular Complications of AIDS.

**Methods:**

We included participants enrolled in the Longitudinal Study of Ocular Complications in AIDS (LSOCA) who completed the Medical Outcome Study [MOS] -HIV questionnaire, the EuroQol, the Feeling Thermometer and the Visual Function Questionnaire (VFQ) 25 every six months thereafter using self- or interviewer-administration. A large print questionnaire was available for participants with visual impairment. Considering all measurements over time and adjusting for patient and study site characteristics we used linear models to compare HRQL scores (all scores from 0-100) between administration formats. We defined adjusted differences of ≥0.2 standard deviations [SD]) to be quantitatively meaningful.

**Results:**

We included 2,261 participants (80.6% males) with a median of 43.1 years of age at enrolment who provided data on 23,420 study visits. The self-administered MOS-HIV, Feeling Thermometer and EuroQol were used in 70% of all visits and the VFQ-25 in 80%. For eight domains of the MOS-HIV differences between the interviewer- and self- administered format were < 0.1 SD. Differences in scores were highest for the social and role function domains but the adjusted differences were still < 0.2 SD. There was no quantitatively meaningful difference between administration formats for EuroQol, Feeling Thermometer and VFQ-25 domain scores. For ocular pain (VFQ-25), we found a statistically significant difference of 3.5 (95% CI 0.2, 6.8), which did, however, not exceed 0.2 SD. For all instruments scores were similar for the large and standard print formats with all adjusted differences < 0.2 SD.

**Conclusions:**

Our large study provides evidence that administration formats do not have a meaningful effect on repeated measurements of patient-reported outcomes. As a consequence, longitudinal studies may not need to consider the effect of different administration formats in their analyses.

## Background

Patient-reported outcomes (PRO) are measured in studies using information that is provided directly by study participants. Probably most commonly, PROs are used as outcome measures in epidemiologic studies and clinical trials [1-5]. But PROs also contribute importantly to the study participants' profile and are often associated with future health outcomes. For example, health-related quality of life (HRQL) or symptoms such as dyspnea can be strong prognostic indicators [6-8].

PRO instruments are either completed by study participants' themselves (self-administered) or administered by an interviewer. Self-administered PRO questionnaires offer the advantage of not requiring research staff as interviewers and participants to complete the questionnaire at their own pace. It may be offered as a paper- and pencil method both at the study site or at home (mail) or through web-based applications. Interviewer-administered PRO questionnaires are more resource intensive but offer additional control over the quality of the measurement. Interviewers may administer the questionnaires face-to-face or over the telephone. In many epidemiologic studies, both self- and interviewer-administered questionnaires are available to accommodate preferences, physical impairment or literacy of participants [9,10].

In a study, the format of questionnaire administration often varies between participants but it may also vary within participants from one follow-up to another. The evidence on the effects of different administration formats on PRO scores is inconsistent. A number of studies (randomized trials or observational studies) found that the administration format had an effect on PRO scores for some or all of the domains [9,11-19]. In some studies scores indicated less health impairment when PRO instruments were administered by an interviewer. A common interpretation of this phenomenon, which is not entirely understood, is that participants may indicate less impairment when interviewed by research staff as compared to self-administered questionnaires. Some refer to this phenomenon as a social desirability bias [20]. Other studies did not find meaningful differences between administration formats [10,21-23]. If effects of different administration formats exist in epidemiological studies or clinical trials estimates of associations or treatment effects may be affected.

Most studies comparing different administration formats were relatively small and considered only one or two measurements [9,11-19]. The results of these studies are inconsistent and it is uncertain whether such unwarranted effects detected in some methodological studies are also present in a particular epidemiologic study where PRO instruments are administered repeatedly over time. Therefore, our aim was to assess the effects of different administration formats on repeated measurements of patient-reported outcomes in a large cohort of persons with AIDS that completed PRO instruments repeatedly over a long period of time.

## Methods

### Study design and participants

We included all participants enrolled in the Longitudinal Study of Ocular Complications of AIDS (LSOCA). Enrollment started in September 1998 and the data included here were collected through December 31^st ^2009. LSOCA is one of the largest prospective observational studies of persons with AIDS. Study participants have AIDS diagnoses according to the 1993 Centers for Disease Control and Prevention case surveillance definition of AIDS. Over the course of the study, recruitment has been performed at 19 clinical centers across the United States, located in urban areas with sizable HIV-infected populations. The current number of active study sites is 13 [24,25]. In this analysis, we included participants with both incident and prevalent AIDS at the time of enrollment.

The study protocol was reviewed and approved by institutional review boards at each of the participating clinics and the coordinating center. Adult participants have given written informed consent. For adolescents, a Consent Statement was signed by parents or guardians and an Assent Statement signed by adolescents and their parents or guardians. More detailed information about the study protocol, data forms and the study handbook is available on http://www.lsoca.com.

### PRO instruments

At enrollment and every six months thereafter, study participants completed the Medical Outcome Study (MOS)-HIV Health Survey, the EuroQol, the Feeling Thermometer and the Visual Function Questionnaire 25 (VFQ-25). Between 1998 and 2008, the subset of participants with major ocular complications (ocular opportunistic infections and major retinal vessel occlusions) had study visits every three months where they completed the questionnaires. The MOS-HIV has 35 items and scores range from 0 (lowest score) to 100 (highest score) [26,27]. Its development was based on the Short-Form 20 of the Medical Outcomes Study and HIV/AIDS-specific domains were added (energy, cognitive functioning, health distress, health transition and quality of life) to the existing domains (general health perceptions, physical function, role function, role function, social functioning, pain and mental health). One item was added to the pain domain. The MOS-HIV has been used extensively in clinical trials and cohorts studies of patients with HIV/AIDS.

The EuroQol consists of five questions about anxiety/depression, mobility, usual activities, pain/discomfort and self-care [28]. Different combinations of responses (on a 5-point Likert-type scale) for the five dimensions are weighted using preferences identified by the US general population [29] The lowest possible score is -0.594 and the highest is 100. The Feeling Thermometer complements the five questions of the EuroQol and asks participants to rate their health status from 0 (equivalent to the worst imaginable health state) to 100 (equivalent to the best imaginable health state). The Feeling Thermometer has been shown to be a reliable, valid and responsive utility measure for various diseases.

The National Eye Institute VFQ-25 was developed to measure vision-specific HRQL in patients with varying eye conditions such as cataract, glaucoma, diabetic retinopathy, cytomegalic virus retinitis and corneal diseases [30,31]. The VFQ-25 measures the influence of visual ability and visual symptoms on health domains and on task-oriented domains. There are domain scores for social functioning, role limitations, dependency on others, mental health, future expectations on vision, near vision activities, distance vision activities, driving difficulties, pain and discomfort in or around the eyes, limitations with peripheral vision and color vision. The VFQ-25 provides reliable and valid scores that are responsive to change. Scores range from 0 (lowest score) to 100 (highest score). In LSOCA, the VFQ-25 was introduced in September 2008.

### Administration formats

The most common format used to complete the HRQL instruments in LSOCA is the self-administered format. This means that participants complete the questionnaires themselves using paper and pencil. Reasons to switch to interviewer-administered questionnaires include inability to read because of sight limitations, dilated pupils for eye examination, illiteracy or for logistical reasons to save time. Thus the choice of administration format depends on characteristics of participants and the study site. The wording and layout of self- and interviewer-administered questionnaires was identical.

In addition, a large print version for all questionnaires was added in May 2008. Participants can complete the large print version if they desire. The font size of the large print version is 14 points compared to 10 points in the standard version. The reasons to switch to a large print format usually relate to the participant's visual impairment or failure to bring reading glasses to a visit. The choice of administration format is made at every visit. Theoretically, the administration format may change from visit to visit although this is rarely the case. The questionnaire administration format is recorded for every visit. For the current analyses, only data from in-person visits were included whereas data from telephone interviews were not considered.

### Statistical analysis

We first determined the number and proportion of interviewer- versus self-administered and large-versus small-print questionnaires, respectively, at baseline and follow-up visits and assessed how these numbers changed as a function of time from enrollment. We also determined the number of participants who switched from the standard self- to an interviewer-administered questionnaire. We calculated mean scores for all HRQL domains stratified by administration ("Proc Univariate" command). We then compared the HRQL scores between administration formats (interviewer- versus self-administered and large- versus small-print) to assess whether they differed, which we defined as ≥0.2 standard deviations from the baseline assessment. The standard deviations for the different instruments and their domains at baseline as well as our thresholds for a quantitatively meaningful difference are shown in Table 1. For each patient, we considered all measurements and administration formats used over time and employed linear regression models ("regress" command of Stata) while accounting for within subject correlation ("cluster" option) and calculating robust standard errors using the Huber-White sandwich estimators ("robust" option). Since the choice of administration format is not random as explained above, patient and study site characteristics are likely to be associated with differences between HRQL scores of different administration formats. Therefore, we adjusted the comparison for study site and the participants' sex and for the time-varying variables age, CD4+ T cells, HIV viral load and visual acuity. We also checked for the potential influence of sex, age and disease severity (CD4+ T cell count) on the effect of administration format and included interaction terms into the regression models to test for effect modification. In a sensitivity analysis, we assessed a cross-sectional sample of participants who switched administration formats from self to interview for the first time. We compared the differences in their mean scores on the two administration formats using the Wilcoxon signed rank test We used SAS (version 9.2, SAS Institute, Cary, NC) for data management and for computing descriptive statistics and Stata for the regression analyses (version 10.1, Stata Corp; College Station, TX).

**Table 1:** Standard deviations for generic and vision specific health-related quality of life scores as obtained from baseline assessment of 2,261 participants enrolled in the Longitudinal Study of Ocular Complications in AIDS (LSOCA)

Generic instruments				Vision-specific instruments			
**Instrument and domain**	**Standard deviation**		**0.2 of pooled standard deviation (defined here as meaningful difference)**	**Instrument and domain**	**Standard deviation**		**0.2 of pooled standard deviation (defined here as meaningful difference)**
	**Self**	**Interview**			**Self**	**Interview**	

**MOS-HIV**				**VFQ-25**			
General health	21.7	23.2	4.5	Composite score	14.0	17.6	3.0
Physical function	26.8	27.9	5.4	General vision	16.0	21.1	3.4
Role function	45.0	45.0	9.0	Ocular pain	18.5	18.3	3.7
Social function	28.6	31.8	6.0	Near activities	19.2	22.2	4.0
Cognitive function	24.2	25.1	4.9	Distance activities	16.6	19.7	3.5
Pain	27.0	28.3	5.5	Social functioning	14.3	17.9	3.0
Mental health	14.7	16.2	3.1	Mental health	19.4	21.5	4.0
Energy	22.1	24.8	4.6	Role difficulties	24.9	26.7	5.1
Quality of life	21.0	22.9	4.4	Dependency	18.1	20.9	3.7
Health transitions	23.8	24.4	4.8	Driving	20.9	31.5	4.7
**Health utility**				Color vision	12.9	17.4	2.8
Feeling thermometer	19.2	21.0	4.0	Peripheral vision	21.3	24.2	4.4
EQ-5D	0.17	0.19	0.036				

## Results

We included 2,261 participants in the analysis. The patient population was predominantly male (81%) with a median age of 43 years (interquartile range [IQR] 38-49), of non-hispanic white (46%) or black ethnicity (36%). At enrollment, 409 participants (18%) had been diagnosed with AIDS for one year or less (incident AIDS) and 1,852 participants (82%) for more than a year. Median CD4+ T cell count at enrollment was 174 cells/μL (IQR 61-339), median nadir CD4+ T cell count was 31 cells/μL (IQR 10-91) and median HIV RNA (viral load) level was 2.9 (log_10_[copies/mL], IQR 1.9-4.7). Overall, 83.0% of participants received HAART at enrollment.

### Administration formats

**Figure 1:**
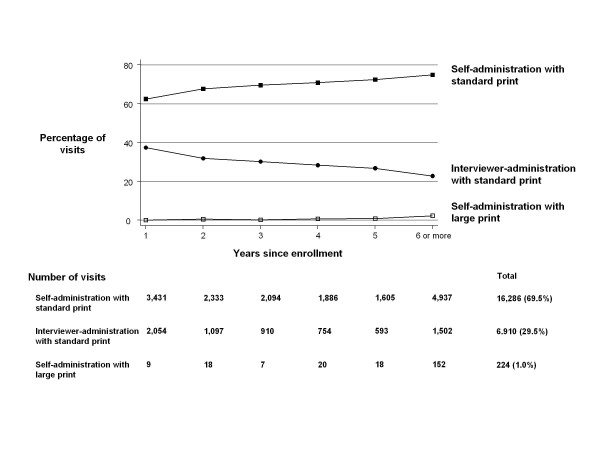
**Study participants and administration formats**. The graph shows the percentage of study participants and the different administration formats they chose since time of enrolment. All study visits (n = 23,420) of all participants (n = 2,261) contributed to the analyses. The percentage of self administration with the standard print increased from 62% in the first year of enrolment to 75% if participants were enrolled six years or more. Interviewer administration with standard print decreased from 37% to 23% and self administration with large print increased from 1% to 2%.

The majority of visits involved self-administered PRO questionnaires (70% of a total of 23,420 study visits). Of the 2,261 patients, 929 (41%) completed their first (baseline) questionnaires via interview and 1,332 (59%) completed it via self-administration. In 6,910 (30%) visits the HRQL questionnaires were interviewer-administered and in 224 (1%) visits participants used the self-administered version with large print letters. These percentages changed with follow-up (Figure 1). The percentages of self-administered questionnaires (standard and large print formats) increased from 63% in the first year of enrollment to 77% beyond five years of enrollment. Of a total of 2,336 visits where the VFQ-25 was completed, participants used the self-administered format in 1,878 (80%) visits (standard print in 1,708 [91%] visits and large print in 170 [9%] visits) and had it interviewer- administered in 458 (20%) visits.

Out of the 2,261 participants, 1,730 (77%) started with the self-administered MOS-HIV, EuroQol and Feeling Thermometer whereas 531 participants (23%) started with the interviewer-administered format. 1,265 (56%) never switched the administration format of the MOS-HIV, EuroQol and Feeling Thermometer, 335 (15%) switched permanently and 661 (29%) switched intermittently. Of the 1,096 participants who completed the VFQ-25 989 (90%) never switched administration format, 93 (9%) switched permanently and 14 (1%) switched intermittently.

### Interviewer- versus self-administered questionnaires

For eight domains of the MOS-HIV, we did not find statistically significant differences between the interviewer- and self- administered formats (Table 2). For the general health perceptions, role function and social function domains, scores were higher for the self- administered format but adjusted differences were < 0.2 SD. The difference between self- and interviewer-administered questionnaires was statistically significant for the Feeling Thermometer but also < 0.2 SD. For the VFQ-25, there was no significant (adjusted) difference for eleven of the twelve domains (Table 3). For ocular pain, we found a significant difference of 3.5 (95% CI 0.2, 6.8) but this difference was below the threshold of 0.2 SD that we defined to be quantitatively meaningful. Unadjusted results were similar to adjusted results for the MOS-HIV, Feeling Thermometer and EuroQol with all differences between administration formats < 0.2 SD. For the VFQ-25, we found differences ≥0.2 SD for six out of twelve domains. We did not find any evidence for an interaction of sex, age and disease severity (CD4+ T cell count) with administration format (none of the interaction terms with p ≤ 0.05).

**Table 2:** Generic health-related quality of life scores: Interviewer- versus Self-administration and Large- versus Small print

Health-related quality of life domain		Interviewer- versus Self-administration			Large versus Standard print format		
	Total (23,420 visits)	Interview (6,910 visits)	Self (16,510 visits)	Adjusted difference* (95% CI)	Large (224 visits)	Standard (16,286 visits)	Adjusted difference* (95% CI)
**MOS-HIV Health Survey**							
**General health perceptions, mean**	63.9	62.1	64.7	-1.7 (-3.2, -0.1), p = 0.03	65.5	64.7	-0.2 (-3.6, 3.2), p = 0.9
**Physical function**	71.2	69.4	72.0	-1.6 (-3.4, 0.3), p = 0.1	68.0	72.0	-3.3 (-7.4, 0.9), p = 0.1
**Role function**	52.1	46.7	54.4	-6.8 (-9.9, -3.7), p < 0.001	54.7	54.4	-2.6 (-9.5, 4.3), p = 0.5
**Social function**	74.9	72.2	76.1	-3.9 (-5.8, -2.1), p < 0.001	72.9	76.1	-0.6 (-5.0, 3.8), p = 0.8
**Cognitive function**	76.6	77.0	76.4	-1.1 (-2.6, 0.4), p = 0.2	75.0	76.4	-0.2 (-3.7, 3.4), p = 0.9
**Pain**	67.0	66.6	67.2	0.5 (-1.3, 2.4), p = 0.6	62.4	67.3	-3.4 (-7.5, 0.7), p = 0.1
**Mental health**	43.3	43.8	43.2	0.0 (-0.9, 1.0), p = 0.9	44.0	43.2	0.8 (-1.6, 3.3), p = 0.5
**Energy**	56.4	54.6	57.2	-0.7 (-2.4, 1.0), p = 0.4	54.6	57.2	-2.8 (-6.6, 1.0), p = 0.2
**Quality of life**	66.3	65.1	66.8	-0.5 (-1.9, 1.0), p = 0.5	65.7	66.8	-3.1 (-6.6, 0.5), p = 0.09
**Health transition**	59.8	59.1	60.1	-0.2 (-1.5, 1.2), p = 0.8	59.8	60.1	-1.2 (-4.7, 2.3), p = 0.5
**Health utility**							
**Feeling Thermometer**	73.8	72.7	74.2	-1.4 (-2.7, -0.1), p = 0.03	75.3	74.2	1.4 (-1.5, 4.3), p = 0.3
**EuroQol - 5D**	0.80	0.79	0.80	-0.01 (-0.02, 0.01), p = 0.3	0.78	0.80	-0.02 (-0.05, 0.01), p = 0.1

* Adjusted for study site, sex, current age, and time-varying covariates CD4+ T cells, HIV viral load, and visual acuity.

**Table 3:** Vision-related health-related quality of life scores: Interviewer- versus Self-administration and Large- versus Small print

Health-related quality of life domain		Interviewer- versus Self-administration			Large versus Standard print format		
	Total (2,336 visits)	Interview (458 visits)	Self (1,878 visits)	Adjusted difference* (95% CI)	Large (170 visits)	Standard (1,708 visits)	Adjusted difference* (95% CI)
**Visual Functioning Questionnaire**							
**Composite visual functioning, mean**	86.5	83.9	87.2	-0.1 (-2.6, 2.5), p = 0.9	87.4	87.1	2.1 (-1.1, 5.2), p = 0.2
**General vision**	76.7	73.2	77.6	-1.8 (-4.6, 1.0), p = 0.2	78.1	77.5	2.2 (-1.6, 6.1), p = 0.3
**Ocular pain**	86.4	87.6	86.1	3.5 (0.2, 6.8), p = 0.04	86.9	86.0	2.3 (-2.0, 6.6), p = 0.3
**Near activities**	83.1	81.5	83.5	1.2 (-2.3, 4.7), p = 0.5	82.6	83.6	2.9 (-1.5, 7.4), p = 0.2
**Distance activities**	88.2	86.1	88.8	0.7 (-2.3, 3.6), p = 0.7	88.0	88.8	2.3 (-1.8, 6.5), p = 0.3
**Vision specific**							
**Social functioning**	93.5	90.3	94.3	-0.7 (-3.2, 1.7), p = 0.6	93.5	94.4	0.9 (-2.8, 4.5), p = 0.6
**Mental health**	84.0	80.8	84.7	-0.4 (-4.1, 3.3), p = 0.8	86.9	84.5	2.7 (-1.7, 7.0), p = 0.2
**Role difficulties**	83.4	80.2	84.2	-2.6 (-7.3, 2.1), p = 0.3	84.0	84.2	0.9 (-4.6, 6.3), p = 0.8
**Dependency**	91.1	87.0	92.1	-1.9 (-5.3, 1.4), p = 0.3	93.4	91.9	2.9 (-1.0, 6.8), p = 0.1
**Driving**	82.6	76.9	83.9	0.2 (-4.2, 4.7), p = 0.9	84.9	83.7	1.6 (-3.2, 6.4), p = 0.5
**Color vision**	95.2	92.5	95.9	-0.3 (-2.6, 2.0), p = 0.8	95.9	95.9	0.6 (-2.8, 4.0), p = 0.7
**Peripheral vision**	87.1	84.6	87.7	1.2 (-2.7, 5.0), p = 0.6	87.6	87.7	3.4 (-2.4, 9.2), p = 0.2

* Adjusted for study site, sex, current age, and time-varying covariates CD4+ T cells, HIV viral load, and visual acuity.

465 participants who started with self-administration and switched at least once to interviewer-administered questionnaires were available for the sensitivity analysis. We did not find any statistically significant differences between scores of the MOS-HIV, Feeling Thermometer, EuroQol and VFQ-25 from the last study visit before the switch (self-administered) to scores obtained at the first visit where interviewer administration was chosen. All differences were below the thresholds for a meaningful difference.

### Large- versus standard print format

For all domains of the MOS-HIV, the Feeling Thermometer and EuroQol the scores were similar for the large and standard print formats and we did not find statistically significant differences (Tables 2 and 3). All differences were below 0.2 SD. Also, we did not find any significant differences for the VFQ-25. Unadjusted differences were also all < 0.2 SD.

## Discussion

In our analysis of more than 23,000 clinic visits of participants with AIDS, different administration formats of generic or disease-specific PRO instruments did not have a meaningful effect on HRQL scores measured repeatedly over time. Differences between all scores of the interviewer- and self-administered questionnaires were below our predefined threshold for a quantitatively meaningful difference. Also, the use of the large print format did not have an impact on HRQL scores.

We defined a meaningful difference between administration formats to be ≥0.2 SD, which corresponds to a small but potentially important difference as first defined by Cohen [32]. Other studies used similar criteria for defining a threshold for meaningful differences between PRO scores [9]. Adjusted differences were all below 0.2 SD, but it should be noted that the estimates were precise for the comparison of the interviewer- and self-administered HIV-MOS with confidence intervals that were mostly within ± 0.2 SD. In contrast, since the VFQ-25 and the large print format were introduced more recently, sample size was considerably smaller for these comparisons and some 95% confidence intervals overlapped ± 0.2 SD. Hence, although mean differences were small for most comparisons of the VFQ-25 and large print format, we cannot claim equivalence of scores measured by interviewer- and self-administered questionnaires. We did not calculate sample size requirements for our study. But if a randomized trial was planned to compare administration formats and the "General Health" domain of the HIV-MOS was the outcome of interest, 526 patients would be needed per trial arm to detect a difference of at least 0.2 SD (4.5 points, assuming a pooled SD of 22.5 points) and a standard 5% chance of two-sided type I (false positive) error and 90% power. Our study sample far exceeded that sample size.

The results of studies comparing different administration formats, including ours, are heterogeneous. Some studies found scores indicating less impairment with interviewer- than with self-administered questionnaires [9,11-19], which was more pronounced for mental health domains in some studies [9,13]. In other studies, investigators did not observe such differences [10,21-23]. To our knowledge, our analysis is the only one embedding the comparison of administration formats in a large cohort study with repeated measurements over time. This allows us to estimate differences between administration formats with greater precision, in a cohort study setting and to do a sensitivity analysis that compared PRO scores within participants. A possible explanation for the absence of differences between administration formats could be that a social desirability bias, that is commonly proposed to explain why interviewer administration leads to higher scores [20], may wash out over time. It seems unlikely, that participants, who come repeatedly for study visits, would consistently overestimate their health. In most studies that compared administration formats, there was only one (cross-sectional) administration where patients are likely to be unfamiliar with the study or clinic setting and where a social desirability bias may be more likely to be present than in a study with follow-up. However, the hypothesis that the social desirability bias washes out over time would require further testing in a randomized trial comparing administration formats where repeated measurements are available.

If an effect of administration format is present investigators should be concerned with a potential effect that may alter inferences in two ways. First, different administration formats may introduce additional measurement variability, which makes the detection of small but important associations or effects more difficult. Sample size requirements to detect a certain difference in PRO could be larger if different administration formats are used because of greater standard deviations and because effect estimates are likely to be attenuated by additional (non-differential) measurement error [9,10]. Second, if different administration formats influence scores, effect estimates could be affected.

**Figure 2:**
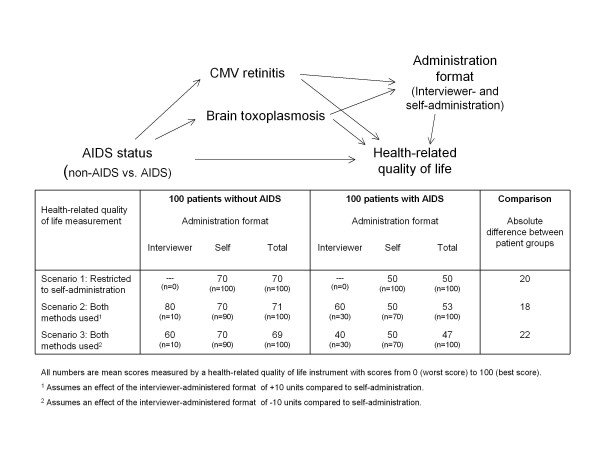
**Relationship of administration format with exposure, outcome and other variables**. A hypothetical scenario is represented by a causal diagram. Investigators may be interested in comparing health-related quality of life (HRQL) between HIV-infected patients with and without AIDS. Both interviewer and self-administration are available. Patients with the AIDS-defining illnesses cytomegalovirus (CMV) retinitis or brain toxoplasmosis are more likely to require interviewer administration because of visual or cognitive impairment, respectively. Administration format is not a confounder since it is on the causal pathway from exposure to outcome and does not cause CMV retinitis nor brain toxoplasmosis. The table shows three scenarios. In the first scenario the administration format is restricted to self-administration and the difference in HRQL is 20 units. In the second and third scenario, both interviewer and self-administration are available and it is assumed that patients with AIDS are more likely to require interviewer administration because of CMV retinitis or brain toxoplasmosis. The effect of interviewer-administration is ± 10 units in the second and third scenario, respectively, which has an effect of ± 2 units on the between-group comparisons.

For example, one could be interested in comparing HRQL between HIV-infected persons with and without AIDS (Figure 2). Let us assume that HRQL is measured by a HRQL instruments with scores from 0 to 100. In the absence of effects from administration format (scenario 1 in Figure 2), we could, for example, expect a mean score of 50 for persons with AIDS and of 70 for HIV-infected persons without AIDS resulting in a mean difference of 20 units. If interviewer-administered questionnaires are offered it can be expected that more persons with AIDS will choose this format (for example 30%) because they have, on average, more visual (for example because of cytomegalovirus retinitis) and more cognitive impairment (for example because of brain toxoplasmosis) than HIV-infected persons without AIDS (scenarios 2 and 3 in Figure 2). If interviewer-administered questionnaires lead to different scores compared to self-administered questionnaires the difference in HRQL between AIDS and non-AIDS persons would be affected.

We see two solutions to address this issue. One solution would be to restrict the administration format strictly to one mode of administration. Since this may be unrealistic in many studies a second solution would be to record the administration format at each study visits and check for an independent effect of administration format on PRO scores as we did in this study. Intuitively, investigators may think that one should adjust the effect estimate for administration format, which has also been proposed in the literature [9]. However, the causal diagram in Figure 2 shows that administration format does not act as a confounder since it is affected by AIDS status, but as an intermediate. In fact, adjusting for administration format would attenuate or increase the association of AIDS status and HRQL and lead to potential under- or over-estimation. Instead, we propose that the effect caused by administration format in some studies should be corrected by the use of methods to account for measurement error such as regression calibration or multiple imputation [33-35]. The idea of regression calibration is to correct the observed value, which is known or suspected not to represent the true value, using information from repeated measurements or from substudies that yield the true values for some patients (e.g. by sing a reference standard measurement method or some instrumental variable). With the multiple imputation approach, the true values are regarded to be missing and can be imputed using information similar to the information used in the regression calibration approach (repeated measurements or true values from substudy). We would like to point out though that we would not consider the effect of administration format to be a measurement error and its effect on estimation an information bias (bias in effect estimation caused by measurement error) since neither of the different methods of measurement are superior and since no reference standard for PRO exists.

Strengths of our analysis include the large sample size and the repeated administrations of PRO instruments over time. Thereby, our analysis represents the typical cohort study settings for which there is little evidence on the effects of administration formats and we have little reason to assume that the results of our study are specific to patients with AIDS only. Another strength is the adjustment for patient and study site characteristics that could have confounded the comparisons. However, a limitation of our study is the lack of randomization for administration format so that some residual confounding might still be present. Also, since the VFQ-25 and the large print format were introduced rather recently the sample size was smaller for investigating the effects of administration format on VFQ-25 scores or of the large versus standard print.

Our large study provides evidence that administration formats do not have a meaningful effect on repeated measurements of PRO. As a consequence, longitudinal studies may not need to consider different administration formats in their analyses. However, if investigators find an effect of administration format they should not adjust for the administration format but consider using one of the methods available for correcting systematic measurement error.

## Competing interests

The authors declare that they have no competing interests.

## Authors' contributions

MP, AA, MVN, CM designed the study. MP, AA, MVN undertook the statistical analyses. MP wrote the first draft of the manuscript. All authors contributed to and have approved the final manuscript.
